# Non-invasive pediatric cardiac imaging—current status and further perspectives

**DOI:** 10.1186/s40348-022-00153-z

**Published:** 2022-12-28

**Authors:** Meinrad Beer, Björn Schönnagel, Jochen Herrmann, Steffen Klömpken, Matthias Schaal, Michael Kaestner, Christian Apitz, Horst Brunner

**Affiliations:** 1 Department of Diagnostic and Interventional Radiology, University Hospital Ulm, Ulm, Germany; 2 Division of Pediatric Radiology, Department of Diagnostic and Interventional Radiology and Nuclear Medicine, University Hospital Hamburg-Eppendorf, Hamburg, Germany; 3 Division of Pediatric Cardiology, Department of Pediatrics and Adolescent Medicine, University Hospital Ulm, Ulm, Germany

**Keywords:** Non-invasive cardiac imaging, Children and adolescents, Echocardiography, Cardiac CT and MRI, Recent advances, Fetal cardiac MRI, Myocarditis, Congenital heart disease, Pediatric cardio-oncology, Artificial intelligence

## Abstract

**Background:**

Non-invasive cardiac imaging has a growing role in diagnosis, differential diagnosis, therapy planning, and follow-up in children and adolescents with congenital and acquired cardiac diseases. This review is based on a systematic analysis of international peer-reviewed articles and additionally presents own clinical experiences. It provides an overview of technical advances, emerging clinical applications, and the aspect of artificial intelligence.

**Main body:**

The main imaging modalities are echocardiography, CT, and MRI. For echocardiography, strain imaging allows a novel non-invasive assessment of tissue integrity, 3D imaging rapid holistic overviews of anatomy. Fast cardiac CT imaging new techniques—especially for coronary assessment as the main clinical indication—have significantly improved spatial and temporal resolution in adjunct with a major reduction in ionizing dose. For cardiac MRI, assessment of tissue integrity even without contrast agent application by mapping sequences is a major technical breakthrough. Fetal cardiac MRI is an emerging technology, which allows structural and functional assessment of fetal hearts including even 4D flow analyses. Last but not least, artificial intelligence will play an important role for improvements of data acquisition and interpretation in the near future.

**Conclusion:**

Non-invasive cardiac imaging plays an integral part in the workup of children with heart disease. In recent years, its main application congenital heart disease has been widened for acquired cardiac diseases.

## Introduction

Non-invasive imaging has gained a fundamental and increasing role as a routine diagnostic in pediatric and adolescent heart disease. Here, congenital heart disease (CHD) served as a kind of pacemaker for the increasingly broader role of these imaging techniques. Depending on national structural requirements, it might be centered at tertiary hospitals or might be offered in the broad. Typically, non-invasive imaging compasses echocardiographic, computed tomography, and MR imaging.

Current guidelines provide a role of non-invasive cardiac imaging for diagnosis, differential diagnosis, and prognosis. Current hot topics in non-invasive cardiac imaging are improvements in spatial and especially temporal resolution, a decrease in contrast agent amount, and the integration of artificial intelligence (AI). The latter addresses data acquisition and data interpretation, as well as data fusion.

The current article reviews established applications of non-invasive cardiac imaging in children and adolescents with a focus on congenital heart disease. Moreover, current technical innovations such as fetal cardiac MRI imaging and AI-based methods are discussed.

### Background and strategy for literature search

Current guidelines emphasize the role of non-invasive imaging in pediatric cardiac diseases. Echocardiography is the primary imaging method with cardiac CT and MRI as cross-sectional imaging modalities for further analysis. Especially the latter show significant recent technical improvements.

The systematic literature search was performed according to the PRISMA 2020 guidelines [1]. In short, a systematic search of studies in the English language was performed on MEDLINE in June and November 2022 (PubMed, https://www.ncbi.nlm.nih.gov/pubmed/). The search was limited to original human studies in peer review journals with an available abstract. No publication date limits were applied. Our aim was to identify studies assessing the value of imaging methods in children and adolescents with heart disease, with also a focus on emerging imaging techniques. Inclusion criteria in detail were (1) original research, (2) sufficiently large enough patient groups, and (3) focus on a pediatric population.

Initially, article screening was performed by two independent readers (M.S. and M.B.) considering only the title and abstract after the removal of duplicates. Both authors read all titles and abstracts independently. All articles that did not meet the inclusion criteria were excluded, and the remaining articles were chosen for reading of the full text.

After independent reading of the full text, articles fulfilling the inclusion criteria (as mentioned above) were selected. Disagreements were resolved by consensus. Finally, references of included articles were hand-searched to check for further eligible studies.

### Echocardiography

Transthoracic echocardiography represents the non-invasive tool most commonly used in the diagnosis and follow-up of patients with any suspected or known heart disease. It enables the assessment of the morphology and function of the heart and is widely used as a diagnostic imaging modality in pediatric cardiology.

Compared with standard protocols in adult cardiology [2], the echocardiographic evaluation in pediatric patients requires a different approach (the so-called segmental approach), providing additional information on the heart position in the thorax, the atrial situs, the veno-atrial, and the atrio-ventricular connections; the relationship between the ventricles; the ventriculo-arterial connections; and the relationship of the great arteries.

Recent developments in functional echo assessment include *strain imaging* and *three-dimensional echocardiography*.

### Strain imaging

**Fig. 1:**
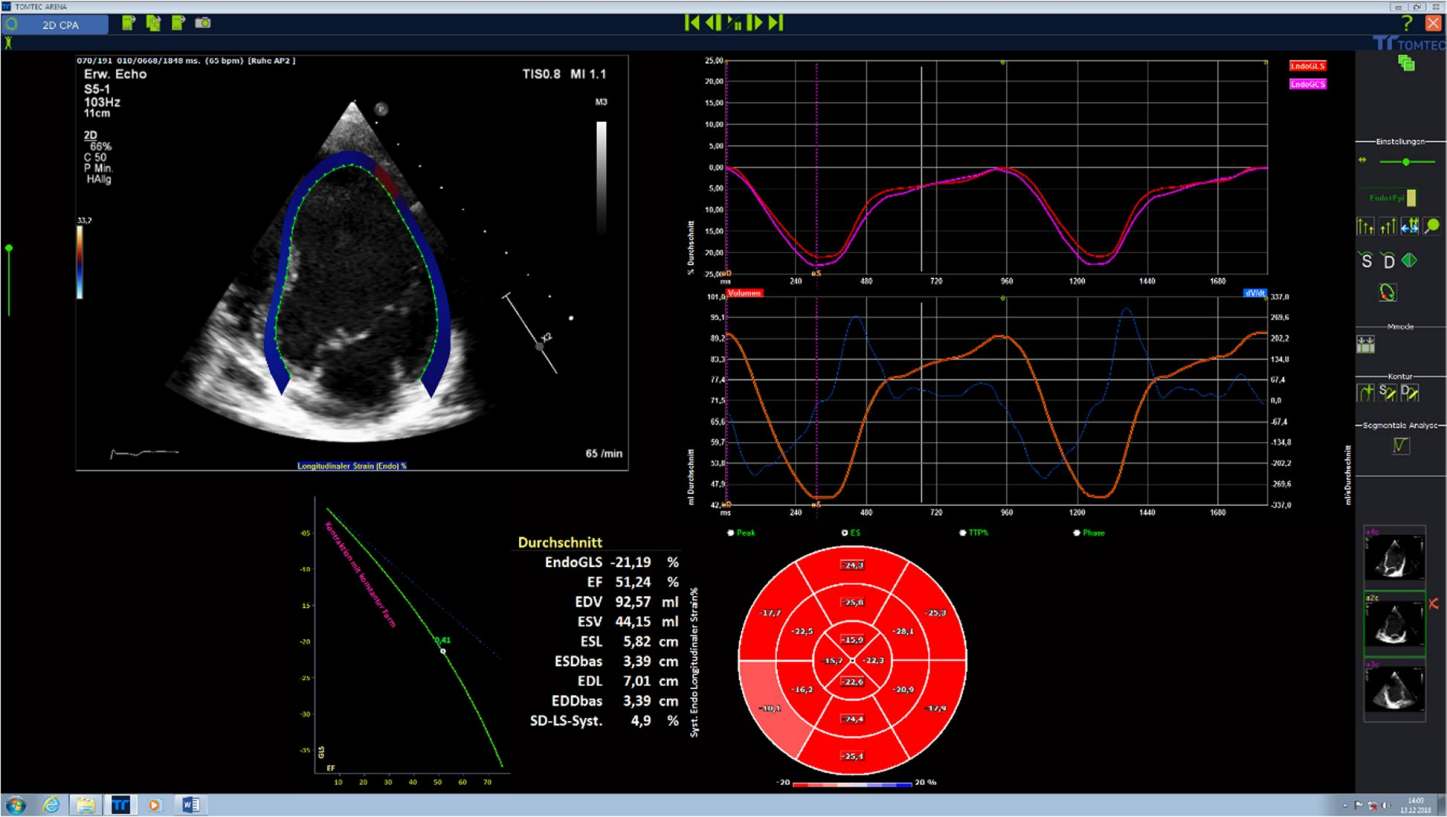
2D strain imaging allows the assessment of global and regional function based on the determination of qualitative and quantitative parameters (longitudinal, radial, circumferential strain): vendor-independent analysis software tools (such as the Tomtec 2D-CPA analysis) visualize LV myocardium, strain curves, and summarize the results in “bull-eye” plots, generating a fast and comprehensive overview on cardiac function

Strain imaging is commonly used for the assessment of ventricular function allowing additional measurement of regional differences in contraction (dyssynergy) or dyssynchrony. Strain imaging (Fig. 1) measures either regional systolic deformation (strain) or the rate of regional deformation (strain rate). The methods used are either tissue Doppler or Speckle tracking echocardiography [3]. Strain imaging can be combined with stress echocardiography (preferably physical exercise) to increase diagnostic sensitivity for early detection of ventricular dysfunction, which might be an interesting diagnostic tool in patient cohorts susceptible for subclinical cardiac dysfunction like patients with congenital heart disease, childhood cancer survivors, or patients with hematologic disease such as thalassemia major [3, 4].

### Three-dimensional echocardiography

Three-dimensional echocardiography (3DE) enables detailed anatomical assessment of cardiac pathology, particularly valvular defects, and cardiomyopathies. The ability to slice the virtual heart in infinite planes in an anatomically appropriate manner and to reconstruct three-dimensional images of anatomic structures makes it unique for the understanding of the congenitally malformed heart [5].

3DE has become important in the management of patients with congenital heart disease, particularly with pre-surgical planning (including 3D printing), to guide the location of bioptomes during right ventricular endomyocardial biopsies and for the guidance of catheter intervention (i.e., placement of catheter-delivered valvular devices) and functional assessment of the heart. 3DE is increasingly used in children because of good acoustic windows and the non-invasive nature of the technique. Novel measurements of 3D deformation, atrial and ventricular volumes, including atrio-ventricular coupling have led to the publication of normal values across a wide range of ages and body sizes [6, 7].

### Cross-sectional imaging: cardiac CT and MRI

Cardiac CT and especially MRI have gained a central role in the diagnosis and differential diagnosis of multiple cardiac diseases in children and adolescents, according to SCMR (Society for Cardiovascular Magnetic Resonance) and other guidelines [8]. Appropriate use criteria for cardiac MRI have recently been published [9, 10]. MRI has tremendous advantages compared to CT, as it allows radiation-free, functional plus tissue integrity assessment beyond mere morphological information compared to CT. Nevertheless, recent technical advancements of cardiac CT have paved the way for CT towards yet unmet applications in the pediatric setting. The transfer of these guidelines into the arena of patient care has to be done next years, which means that standardized cardiac CT and MRI as well echocardiography protocols have to be applied in a broader range.

### CT

In nuce, the major application for cardiac CT in children is the delineation of coronary anatomy. Here, the detection or exclusion of coronary anomalies (high-risk vs. low-risk variants; coronary fistulas) is the major task [11]. For that purpose, ultra-fast protocols are essential [12]. Applications beyond coronaries include assessment of complex congenital heart disease in neonates (depending on the availability of high-end fast and ultra-low-dose CT scanners, see below) and generally in children with contraindications for MRI.

Besides the standard single-source CT scanners (one x-ray-tube, one detector), dual-source high-end scanners (DSCT) are available since almost two decades (two x-ray-tubes, two detectors). Dose reduction and high temporal resolution are advantages of DSCT scanner technology; however, high costs are a major drawback and limit widespread availability. Most recently photon-counting CT technology has been introduced, which might further reduce radiation dose and increase image quality and thus overall diagnostic accuracy [13].

#### Protocols

It is mandatory to use distinctive CT protocols for pediatric examinations. The newest CT scanner generations [14] plus denoising technologies based on AI algorithms [15] are additionally of high importance.

**Fig. 2:**
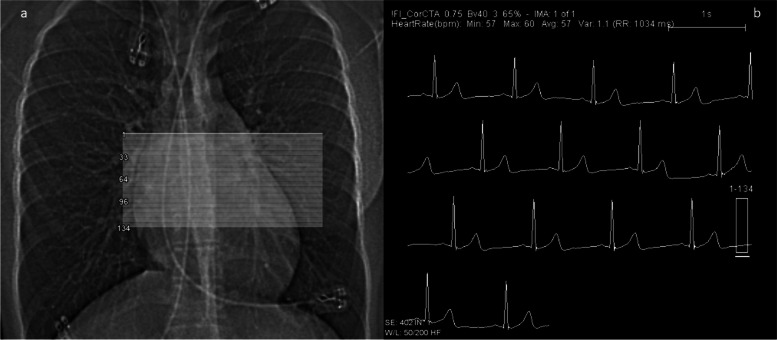
(Planning and dose monitoring): first steps are a fast low-dose overview (“topogram/sinogram”; left) and a stable ECG monitoring (right) for triggering (prospective mode) or gating (retrospective mode). Figures 2 and 3 derive from the CT of a 15-year-old girl after surgical repair of a malignant coronary anomaly (origin of the left main from the right coronary sinus) with reinsertion of the left main coronary artery with an ultra-low radiation dose (total DLP 9 mGycm ~ 0.2 mSv)

**Fig. 3:**
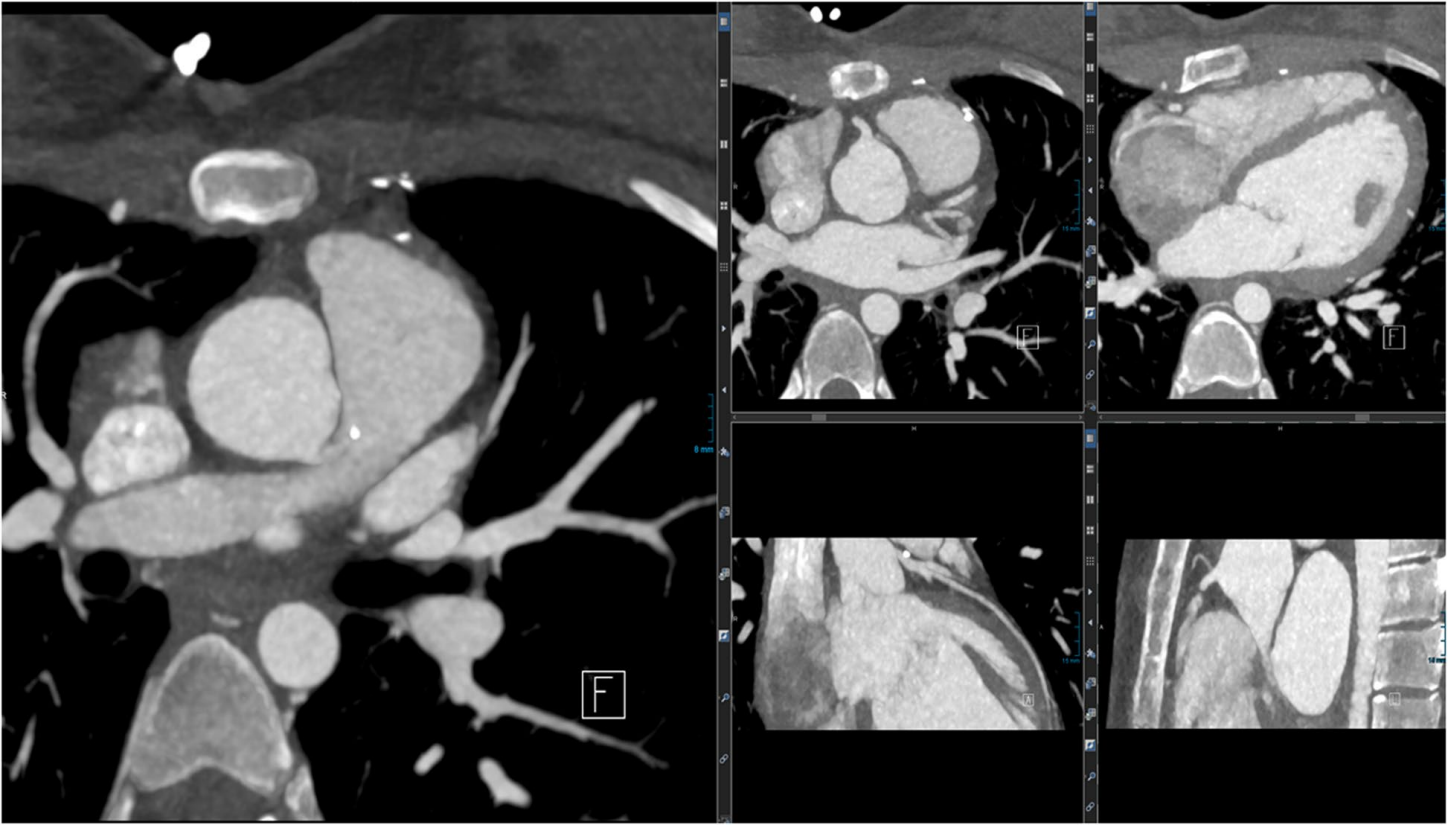
(Representative images for the primary CT datasets): based on primary axial planes (left), different positions in long-axis heart orientation (right upper row) and parasagittal orientation (right lower row) are depicted. Currently, these standard projections are reconstructed by specifically trained technicians; increasingly AI-based algorithms take over these time-consuming tasks

The CT examination encompasses distinct steps, which are summarized in Figs. 2 and 3.

**Fig. 4:**
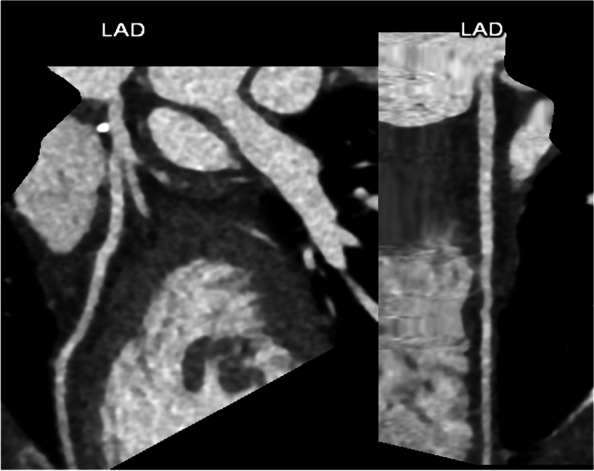
(2D postprocessing): automatically 2D recontructions of the three main coronary arteries are generated, here the LAD in curved (left) and straight (right) MIP projections; this allows a rapid assessment at first sight and facilitates the review of finding by the referring physicians

For the postprocessing, individually arranged image analyses are standard (Fig. 4); additionally orthogonal planes to the aortic valve can be reconstructed. Moreover, coronary and sagittal projections for the heart are processed.

### MRI

The most established application field of cardiac MRI is congenital heart disease [16–19]. Besides this, the assessment of inflammatory diseases (myocarditis) [20] and the emerging field of cardio-oncology (myocardial involvement due to potentially cardiotoxic anti-cancer therapeutics) plus the new field of fetal cardiac MRI [21, 22] are of high importance. Apart from fetal cardiac MRI—which will be presented in more detail later in this article—the borders to examinations of adults become more indistinct compared to previous times as state-of-the-art cardiac MRI techniques have such a high temporal and spatial resolution and integrate free-breathing technologies and can be thus utilized for pediatric as well as adult cardiac MRI examinations.

The semi-automatic character of these new state-of-the-art cardiac MRI techniques (autocalibration to individual geometries, breathhold capabilities, ECG rates; semi-automatic definition of image planes according to the individual heart axis during the examinations; automated postprocessing) in pediatric cardiac MRI is and will be offered on a broader range beyond specialized centers. This will pave the way for on-site assessment of even complex cardiac conditions.

Another rapidly growing field is the fusion of imaging modalities, e.g., echocardiography with cardiac MRI. Especially planning and execution of pediatric cardiac interventions significantly benefit from this kind of comprehensive imaging guidance [23, 24]. Artificial intelligence (AI) is one of the integral parts for image fusion (automatic calibration, online co-registration plus reduction of measurement times [25]) and will be also discussed in more detail later on.

#### Protocols

The basic components of cardiac MRI protocols are cinematographic imaging (CINE), 2D flow and late gadolinium enhancement (LGE), and 2D/3D flow. Extended protocols also include 4D flow and mapping.

**Fig. 5:**
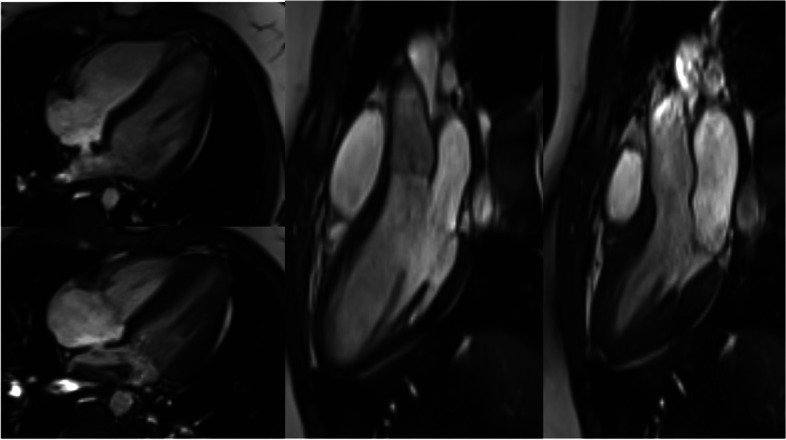
(Functional imaging): assessment of cinematographic (“CINE”) images allows a rapid overview of global and regional functions comparable to echocardiography. Standard are planned projections in the long- and short-heart axis. Here, images of a 12-year-old girl with follow-up after multisystem inflammatory syndrome in children (PIMS) are presented: a 4-chamber view in diastole (left upper row) and systole (left lower row) and a 2-chamber view also in diastole (middle) and systole (right)

Figure 5 summarizes integral parts of the LV and RV analyses.

**Fig. 6:**
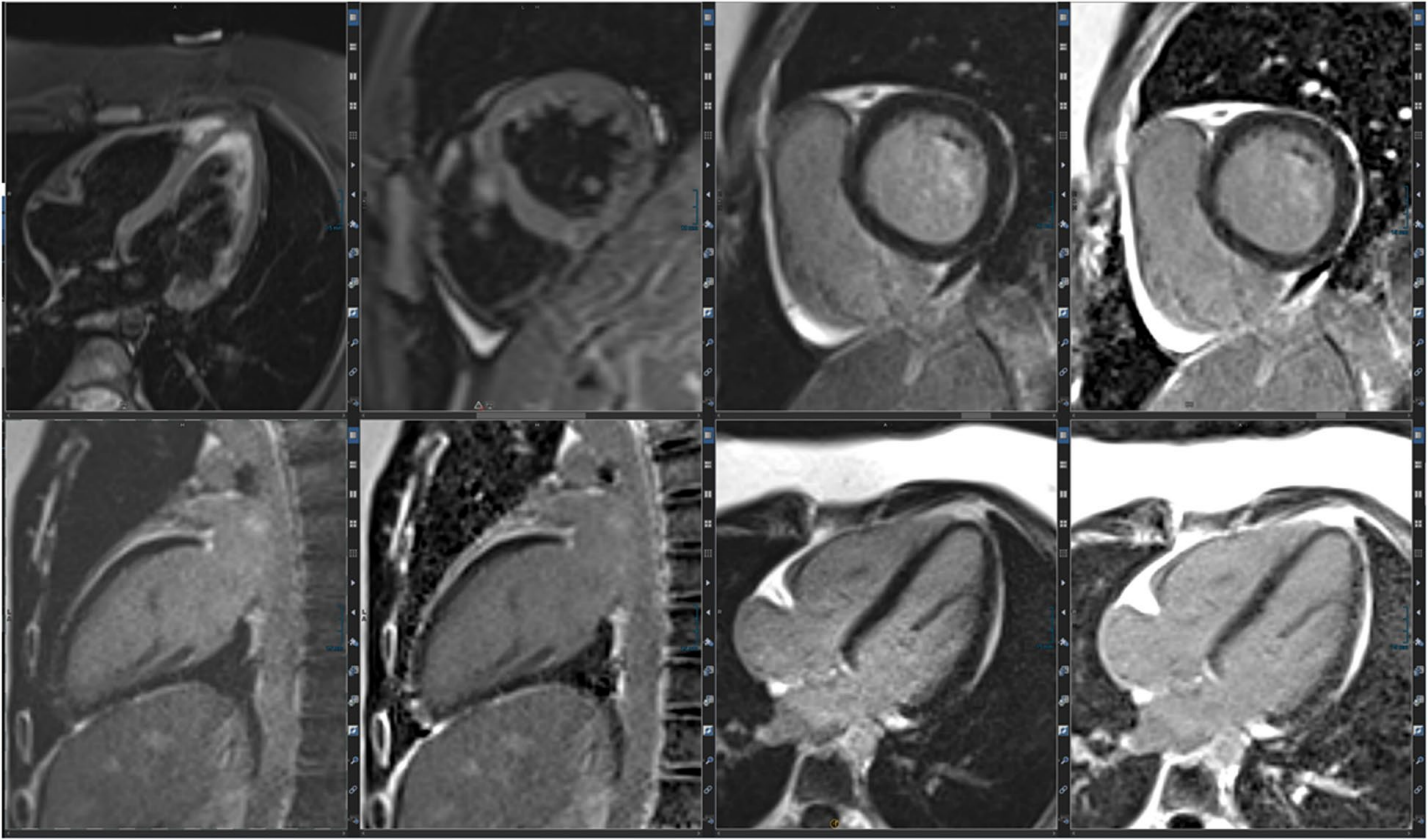
(Tissue integrity assessment by T2 and LGE imaging): the capability of a non-invasive complete assessment of tissue integrity (edema yes/no, fibrosis yes/no) is one of the hallmarks of MRI. Healthy myocardium shows a dark (“hypointense”) signal in T2 imaging (first two images in the left upper row) as well as a dark signal in LGE imaging (right upper row LGE-SAX in IR and PS reconstruction; lower row—LV 2CV- and 4 CV-LGE)

Especially for myocardial inflammation and cardio-oncology assessment of tissue integrity by T2-weighted imaging plays an important role besides LGE (Fig. 6).

T2 images reveal increased water content within the myocardium by increased myocardial signal intensities. Inflammation as well as acute coronary artery disease are accompanied by myocardial edema. This qualitative evaluation can be detected by T2 imaging.

LGE images are typically acquired 10 min after intravenous contrast agent injection. At this time point, gadolinium (Gd) has already been cleared from the myocardium (wash-out processes, which require energy and thus sufficient perfusion and tissue integrity).

#### Mapping technologies and controversy about Gd contrast agent application

**Fig. 7:**
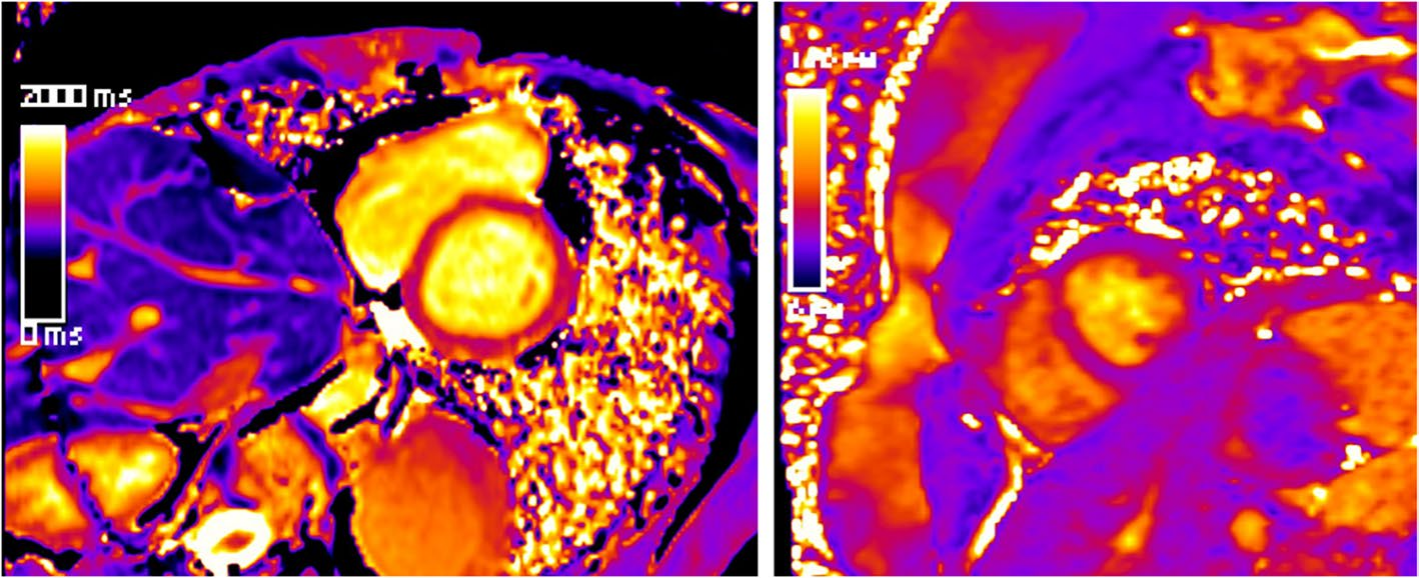
(Mapping technologies): qualitative (based on color maps) and quantitative (based on signal intensities) assessment is possible; in comparison to values of healthy cohorts, increased T2 values indicate myocardial edema and increased T1 values indicate fibrosis; here, healthy myocardium is depicted for SAX-T2- (left) and pre-contrast (“native”) T1 mapping (right) in midventricular position

Mapping techniques are increasingly replacing traditional LGE and T2-weighted imaging (Fig. 7). Their advantages are manifold. Two main aspects are their inherent potential for quantification plus the concomitant effect of reduction of Gd contrast agents. This is important in the ongoing discussion of the potential harmful effects of Gd contrast agents.

Add-ons to standard MRI protocols are MR angiography (MRA) and 2- up to 4D flow analyses. This is especially important in CHD. State-of-the-art MRA techniques cover more than one cycle of contrast agent inflow (time-resolved angiography).

Flow analyses are important in valve disease and in the assessment of shunts. Traditionally, 2D techniques are applied, which allow a fast and robust determination of flow hemodynamics (e.g., volumes, velocities, flow direction including regurgitations), and when applied in orthogonal orientation. Recently introduced 4D flow techniques display a volume (with a future perspective of whole heart coverage). Yet, the latter has to be integrated into routine scanning.

### Current status—from a clinical point of view

#### Congenital heart disease (CHD)

Congenital heart disease (CHD) is the major application of non-invasive imaging, especially for cardiac MRI. This is due to the challenging complex anatomy of CHD, which also affords the assessment of hemodynamic and structural parameters, a specific strength of cardiac MRI. Besides LGE and mapping as surrogates for fibrosis (inverse remodeling, structural changes), 3D or even advanced 4D flow is an essential module.

**Fig. 8:**
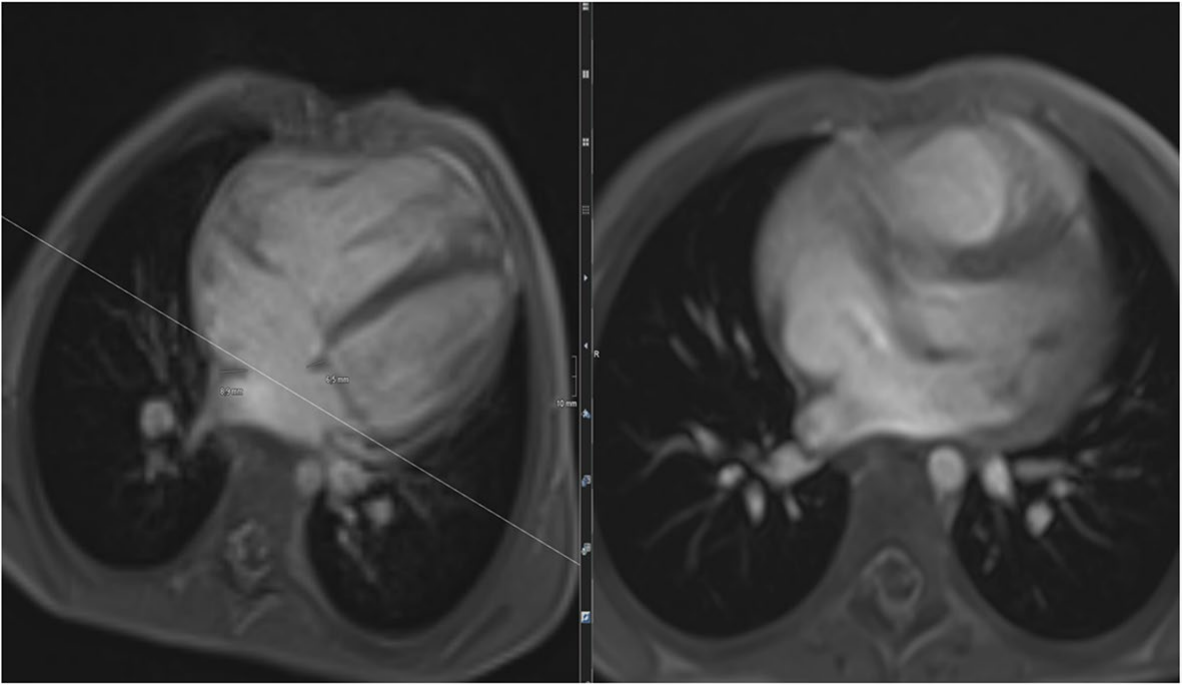
(Functional imaging): a 4-chamber view and axial CINE allow the measurement of the size of the ASD (diameters) plus the illustration of concomitant “jets” (increased and turbulent flow produces hypointense blood signals)

**Fig. 9:**
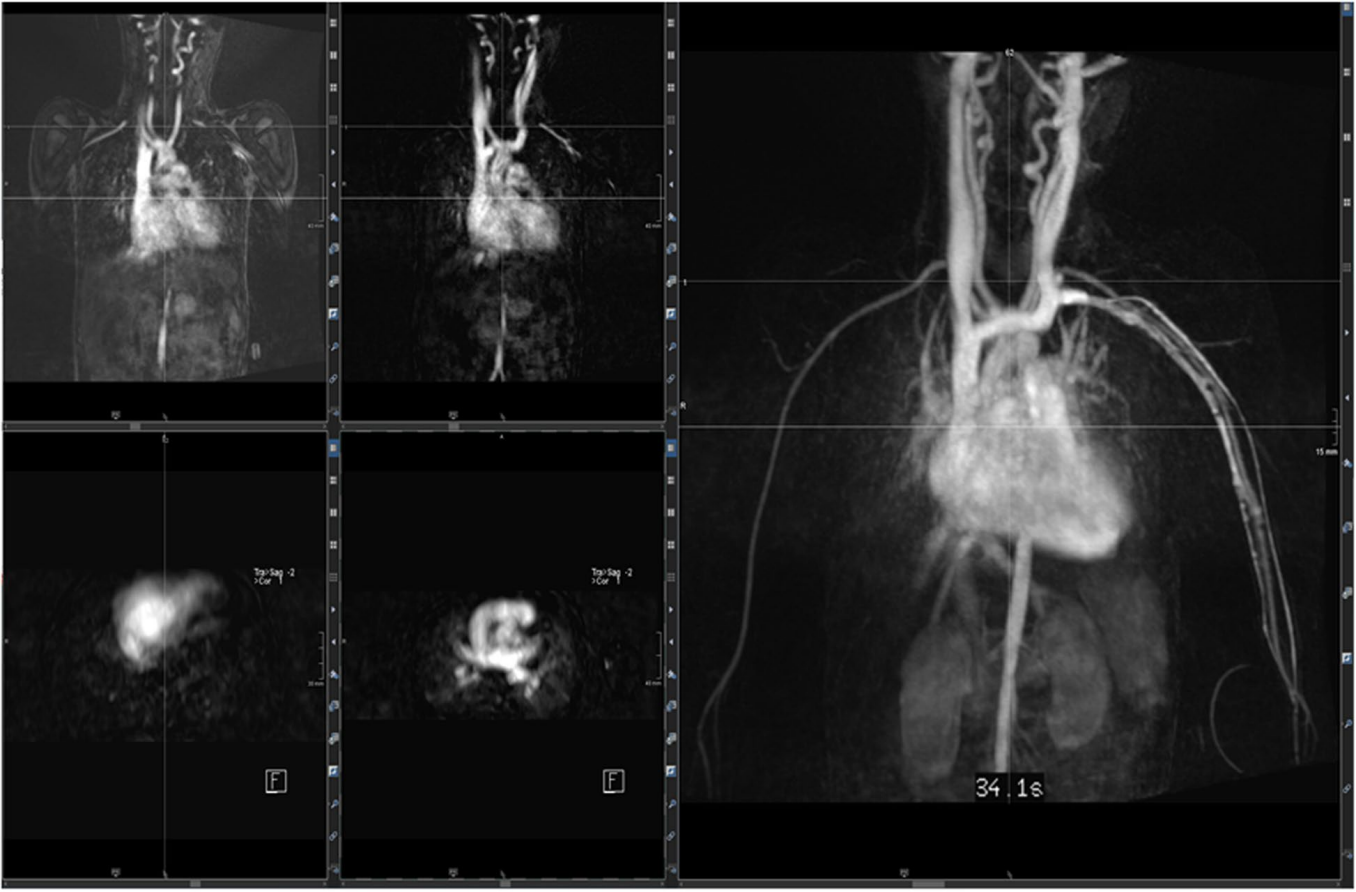
(MRA): time-resolved contrast-enhanced MRA in coronal and transversal reconstructions (MIP projections) allow a complete overview of the anatomy of large vessels and the monitoring of shunting (left-right; right-left)

**Fig. 10:**
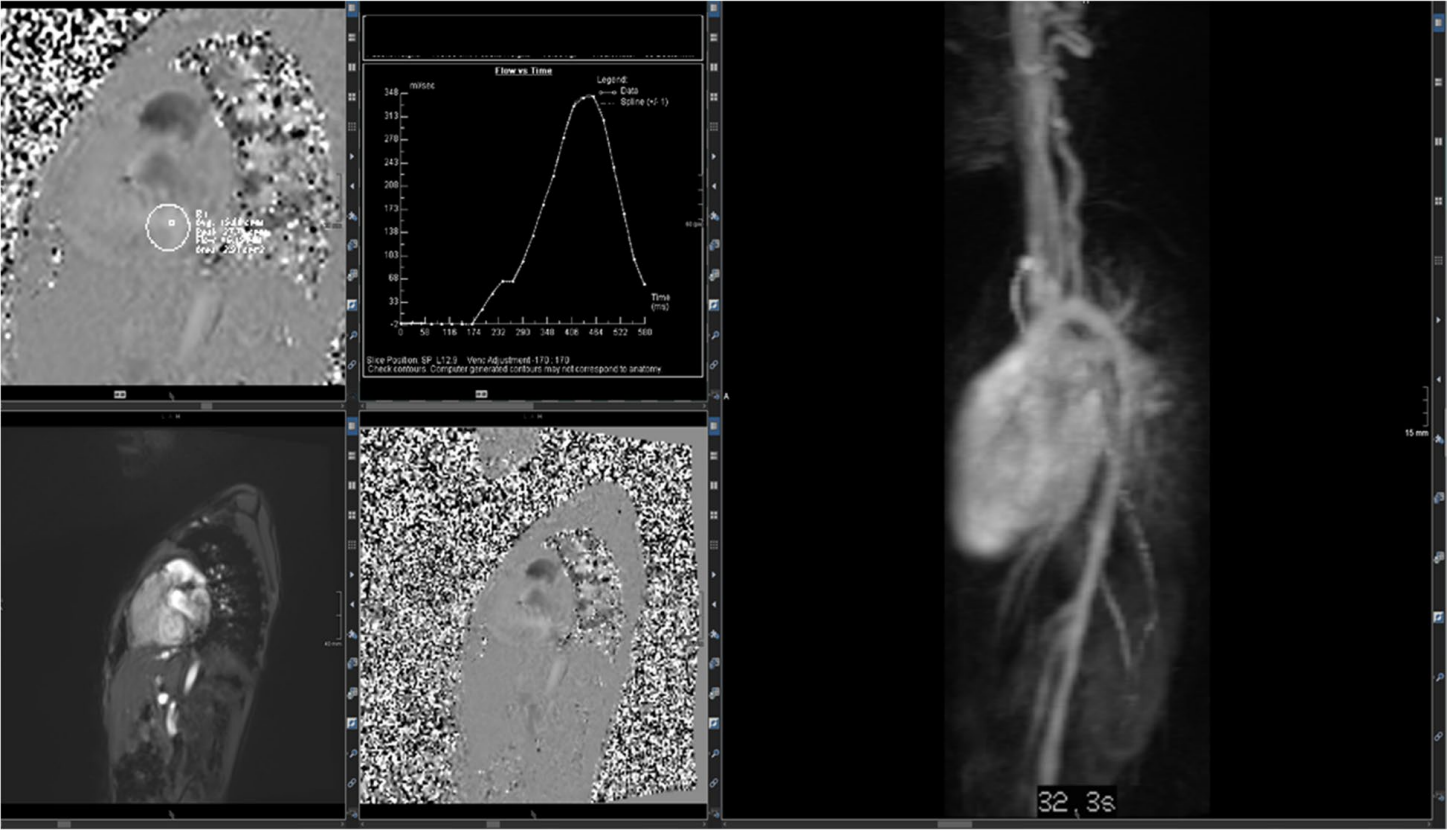
(2D flow): left–right shunting due to the ASD. Flow is depicted morphologically (“magnitude images,” left lower row) and hemodynamically (“phase-images, left upper row and middle lower row”); the latter allows a quantitative assessment of velocities, directions, and flow profiles (upper row in the middle)

A first example for CHD assessment by cardiac MRI is a 6-year-old girl (Figs. 8, 9, and 10) suffering from restricted cardiac function with a large atrial defect (ASD). The 4-chamber view shows a defect of more than 2 cm between the right and left cardiac atrium. Volumetric analysis by CINE MRI demonstrated a left–right shunt volume of 60%, thus classifying the defect as a III° up to IV° defect. Adding flow analysis and contrast-enhanced MRA confirmed the diagnosis and grading. The shunt volume triggered an enlargement of the right atrium and ventricle, without functional impairment. The connection between the four large pulmonary veins into the left atrium was correct, and an additional slight (grad I°) tricuspid regurgitation was noted (17% in phase-contrast imaging).

**Fig. 11:**
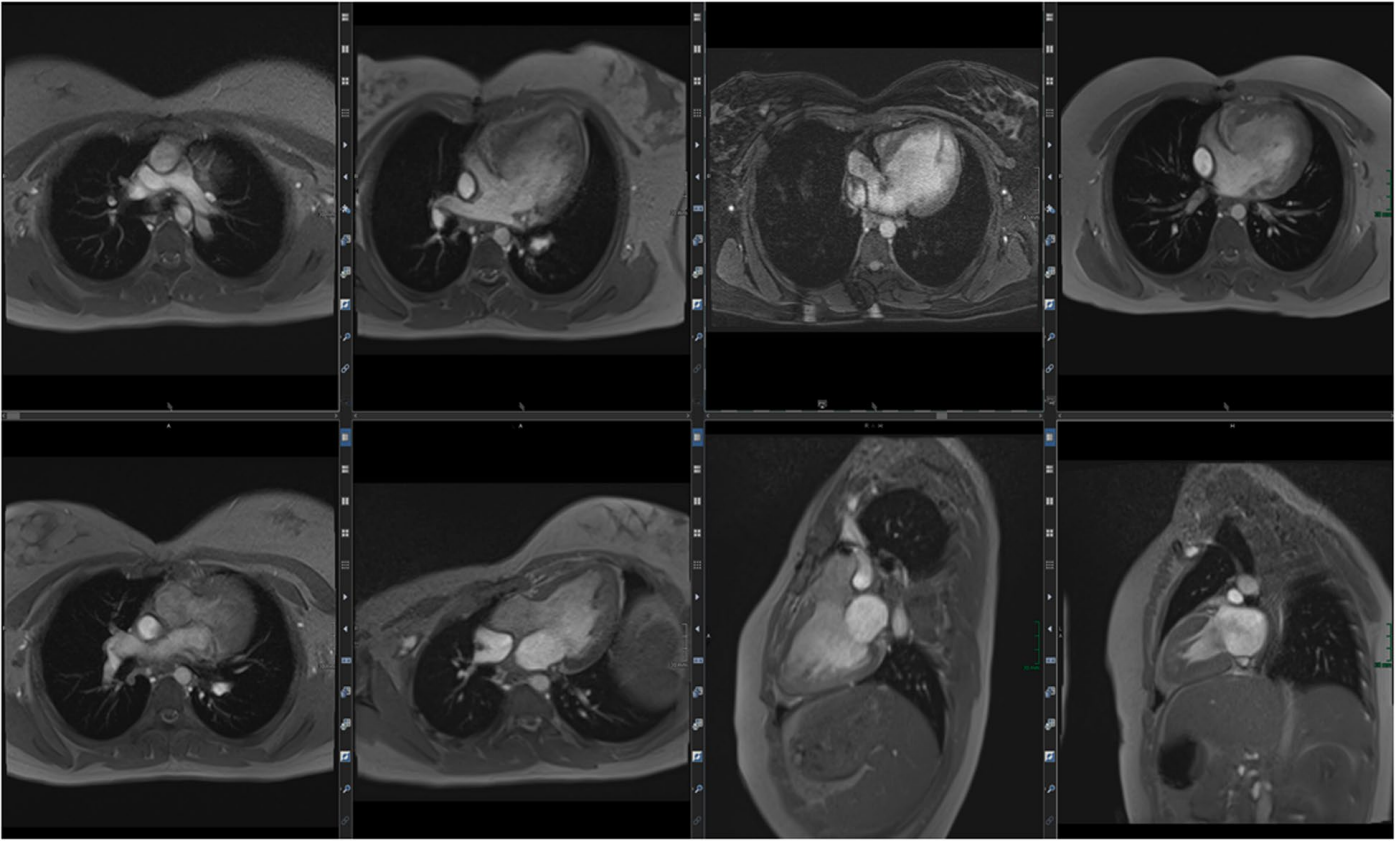
(Functional and anatomic imaging by CINE): 4CV, 3CV, 2CV, and axial CINE show the enlarged and hypertrophied LV as well as the TCPC

**Fig. 12:**
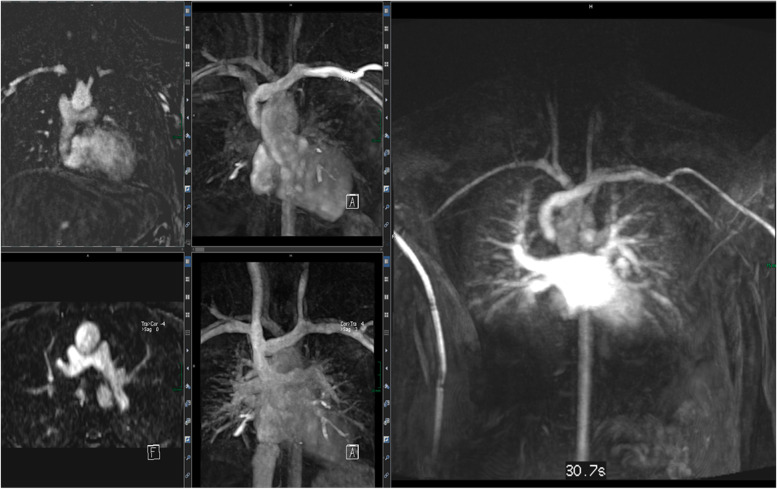
(MRA): connection between VCS, VCI, and right pulmonary artery. The 4 smaller images show the connections (upper and lower images on the left as single images in high resolution; upper and lower images in the middle as thicker MIPs for an overview). The right larger image is one data set of the MR angiography, which gives an idea about pulmonary blood flow

More complex CHD is presented in the case vignette of a 14-year-old girl (Figs. 11 and 12) with a single ventricle after total cavopulmonary connection (TCPC). The right ventricle is only a remnant with a complete atresia of the tricuspid valve. Typically after TCPC, the vena cava superior and inferior mouth into the right pulmonary artery without detection of stenoses. The perfusion of both lungs was almost equivalent with a slight predominance of the left lung (56% left, 44% right). A “atrium commune” was created after atrial septectomy.

The implantation of devices (for pacing etc.) in patients with CHD is an increasing challenge for cardiac MRI. Examinations in children with implanted devices afford close interaction between pediatric cardiology, partially adult cardiology, and pediatric radiology. Expert consensus statements concerning the practical handling are helpful [26].

#### Morphologic alterations beyond CHD—coronary fistulas

**Fig. 13:**
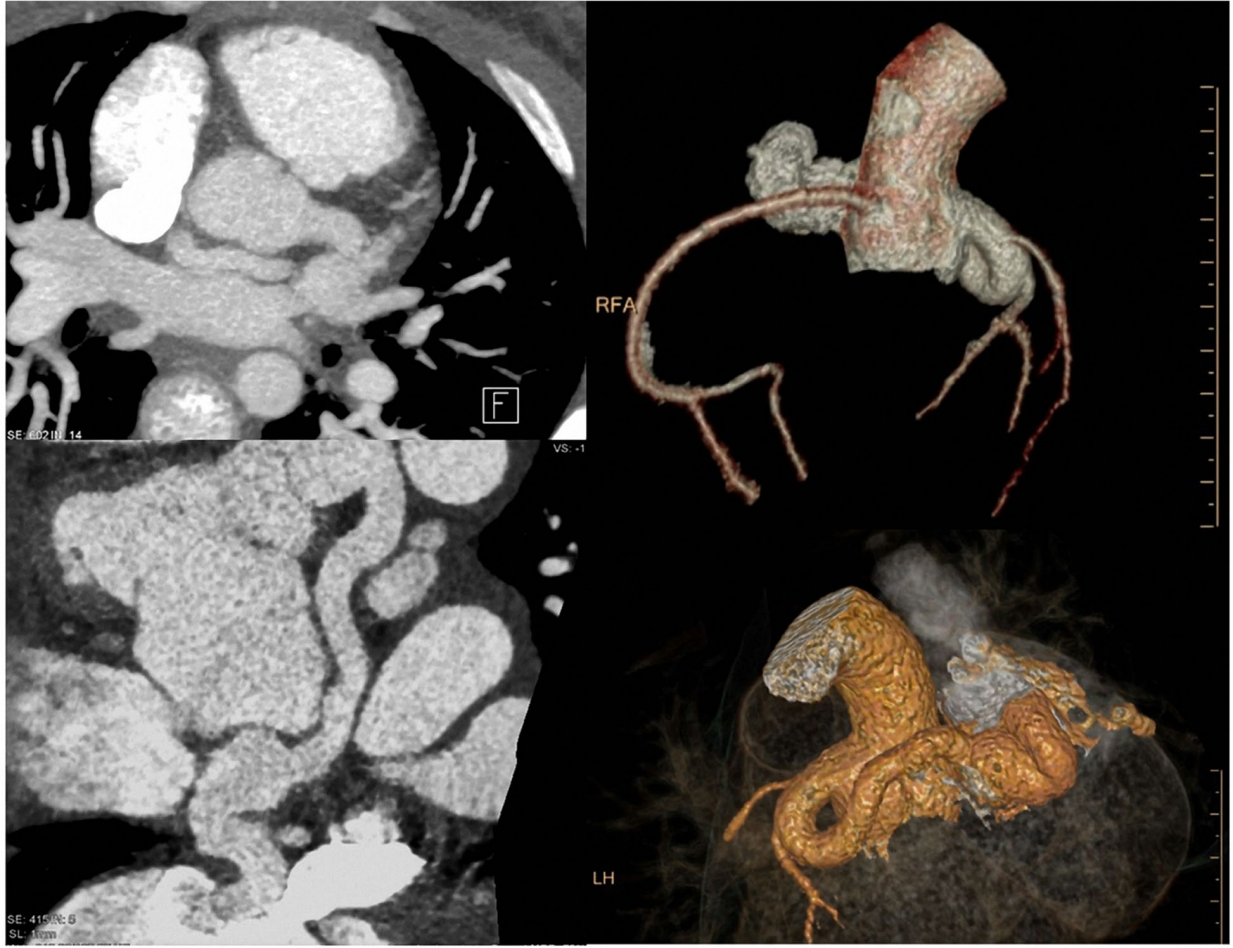
(Planning, dose monitoring (left upper and lower row), axial and paracoronar 2D MIP reconstructions (middle), and 3D VRT reconstructions (right)): 11-year-old girl. Previous surgical repair of a VSD. Now suspected (echocardiography—not shown) coronary fistula. CTCA with dose reduction software, resulting in a low radiation dose (total DLP 63 mGycm ~ 0.8 mSv). CT allows a complete and rapid representation of even highly complex anatomic alterations (in one breathhold)

Coronaries and their assessment are still an unmet need by cardiac MRI. Here, cardiac CT is also in pediatric populations the method of choice for non-invasive assessment. A typical example is an 11-year-old girl (Fig. 13), who was referred to our department due to reduction of cardiac function and palpitations. Non-invasive echocardiography showed an unclear coronary situation with a high suspicion of coronary shunting. In an interdisciplinary discussion, the decision to apply cardiac CT (and not cardiac MRI) was made for this situation. The 3D reconstruction demonstrates a coronary fistula with an enlarged sinus artery and a left–right shunting. The origin was the left main branch with a mouth into the right atrium just lateral the cavo-atrial connection. The radiation dose was below 1 mSv due to the application of dose reduction software (iterative reconstruction) during data acquisition. Minimal-invasive therapy was planned.

#### Inflammation/myocarditis

A leading application of non-invasive imaging is the workup of inflammatory heart disease. Besides the “traditional” viral myocarditis, COVID-19-associated acute as well as chronic myocarditis has developed into a major challenge for clinical care. Imaging, i.e. cardiac MRI, is one of the pillows in clinical decision-making. The Lake Louise 2.0 criteria have made things simpler. A contrast-free CMR assessment is now possible for the diagnosis of myocarditis, integrating T1 native mapping sequences and T2-weighted imaging. Thus, COVID-19 gave some impetus for this development from contrast towards contrast-free cardiac MRI.

**Fig. 14:**
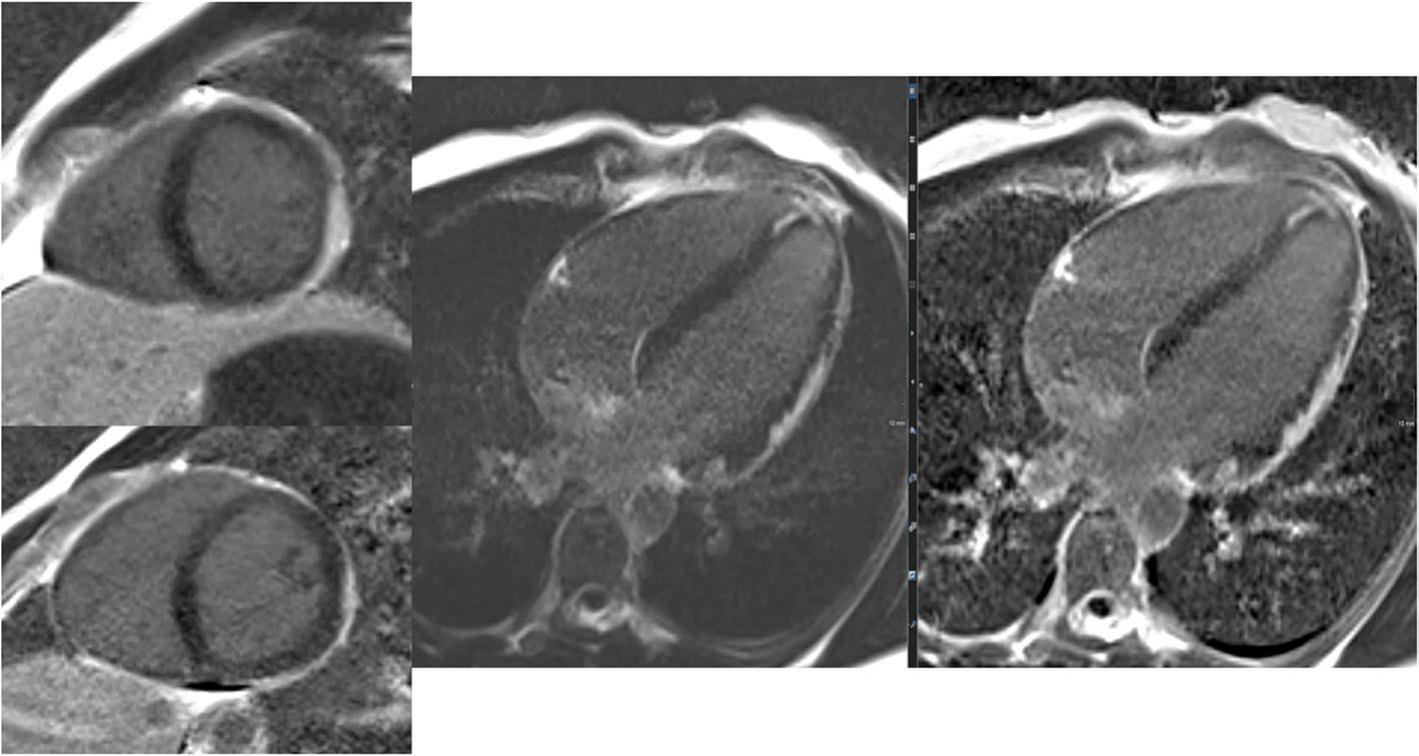
(LGE imaging): SAX (left upper and lower row) and 4-chamber view in IR (middle) and PS (right) reconstructions show an increased subepicardial signal of the left lateral wall, partly including mid-wall parts of the LV myocardium

**Fig. 15:**
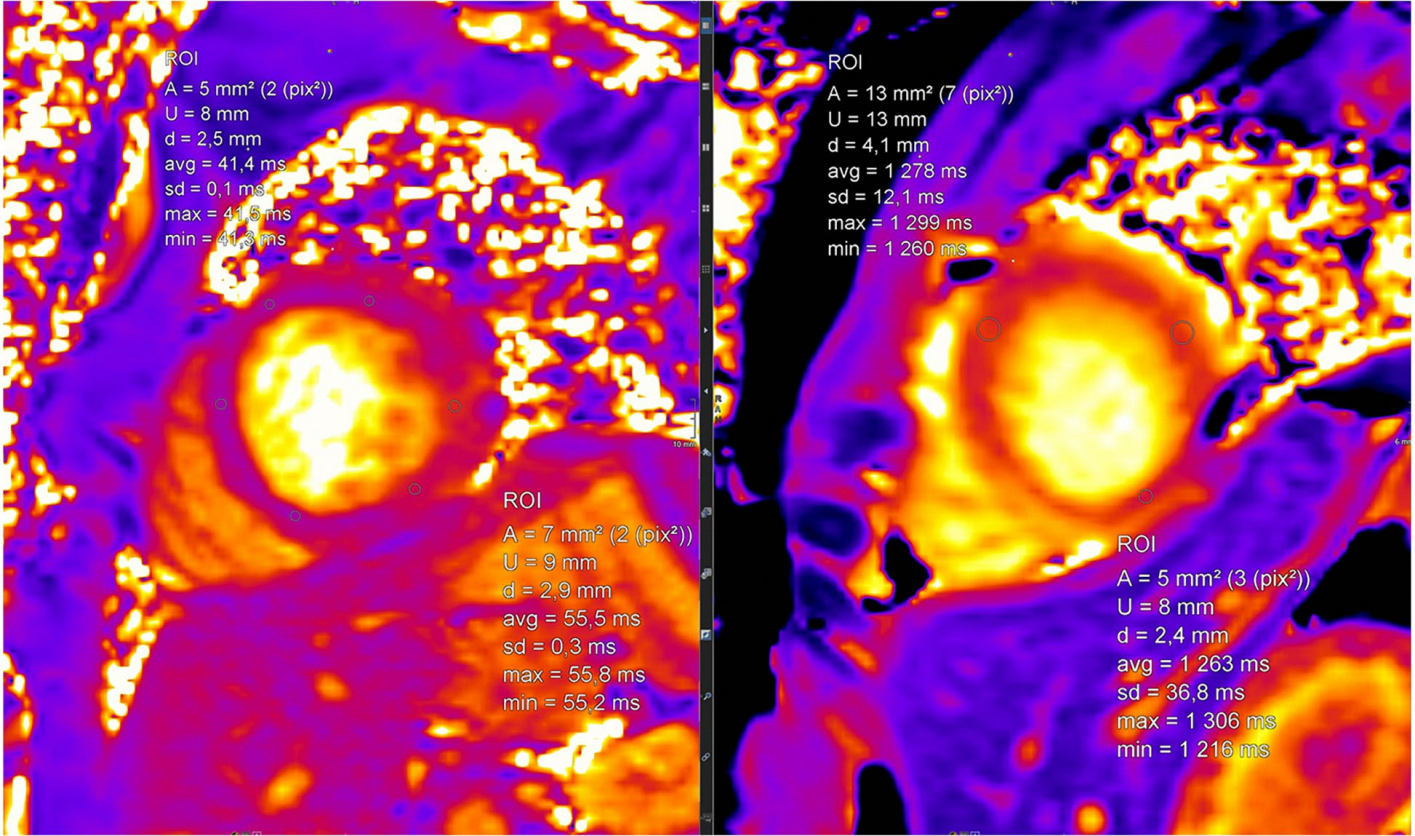
(Mapping): T2 map (left) anteroseptally 37–40 ms; inferolaterally 49–56 ms, T1 map prä KM (right) anteroseptally 1278 ms; inferolaterally 1263 ms. The color maps allow a rapid assessment of edema and inflammation/fibrosis plus the quantification (in regions, which are conspicuous in LGE and/or maps)

A first case vignette (Figs. 14 and 15) presents images from a 17-year-old adolescent with suspected myocarditis. Troponin levels were increased; additionally, ECG showed ST elevations. There was only a slight decrease in global LV function without dilatation and without regional dysfunction. LGE showed extended contrast enhancement and mapping revealed increased T1 and T2 relaxation times especially at the LV myocardium.

**Fig. 16:**
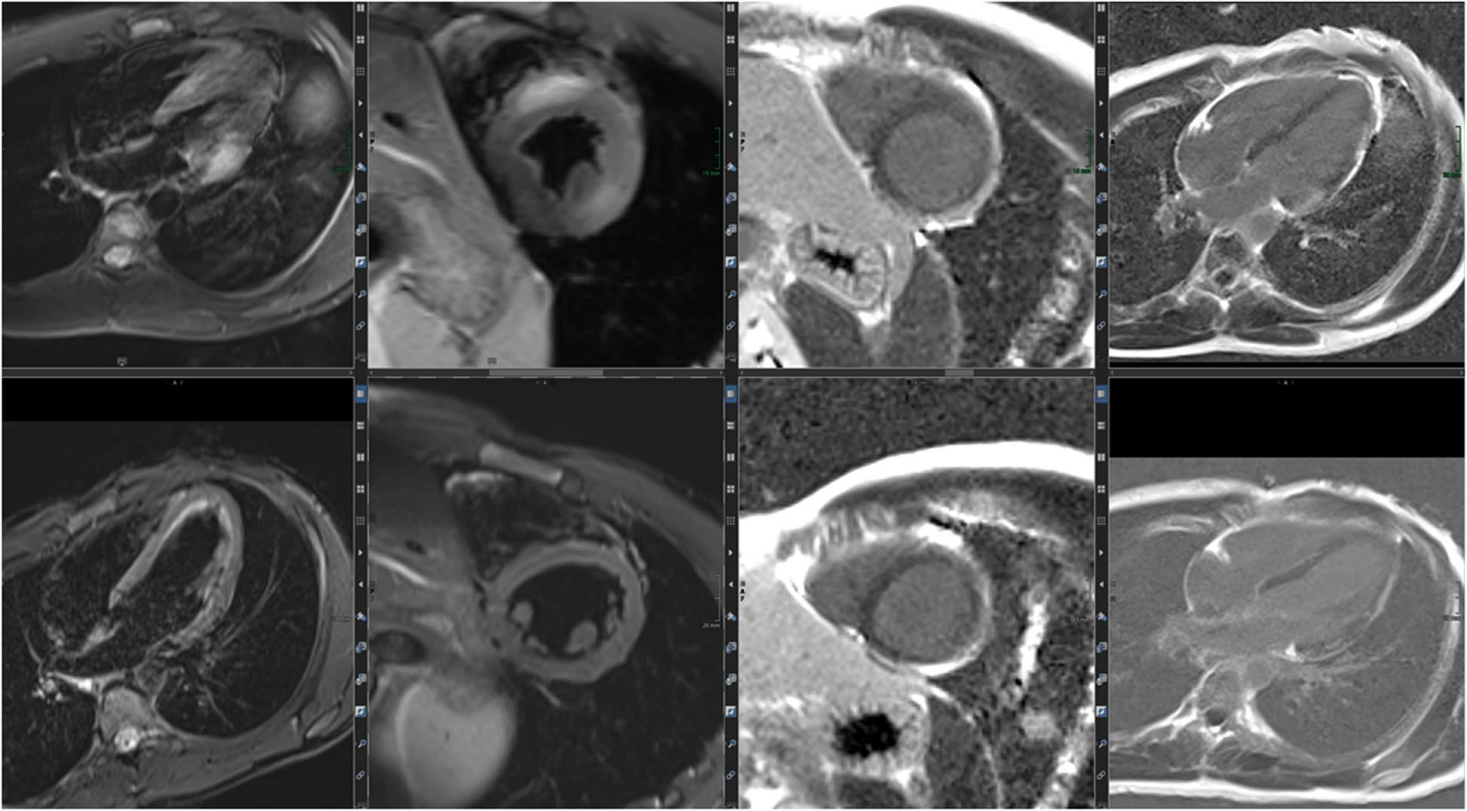
(T2 and LGE imaging during follow-up): upper row—baseline; lower row—follow-up 10 weeks later. Complete normalization of increased T2 signal in septal and lateral myocardium accompanied by a slight decrease of LGE with the typical subepicardial predominance. Loss of edema signal in conjunct with decreased LGE signal suggests an end of the active inflammatory phase with ongoing repair in altered myocardium

**Fig. 17:**
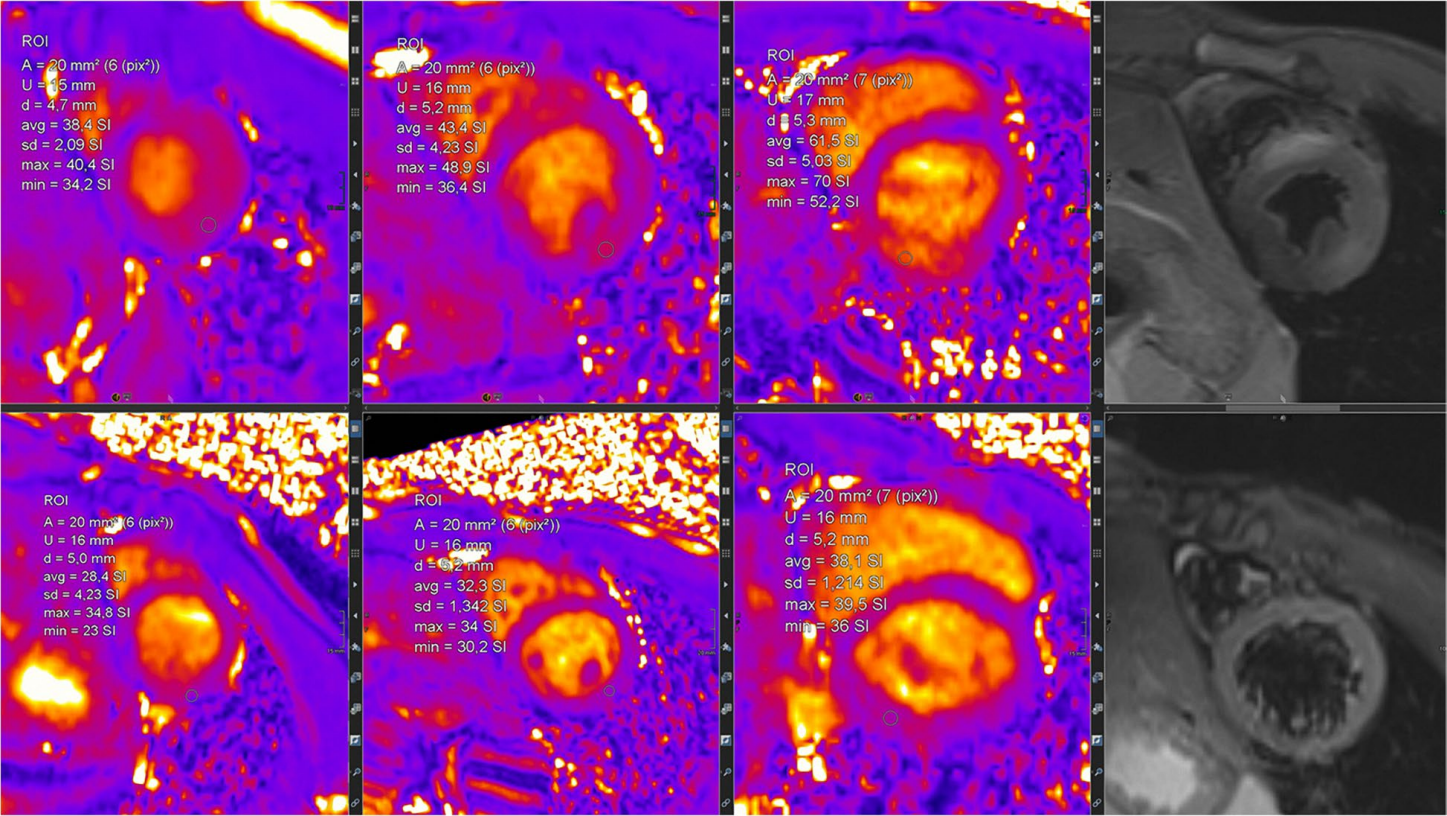
(T2 mapping during follow-up): normalization of initially increased signal intensities in septal LV myocardium (upper row—baseline with 38–62 ms; lower row—follow-up with 28–38 ms). This further supports the mentioned post-acute myocarditis stage

**Fig. 18:**
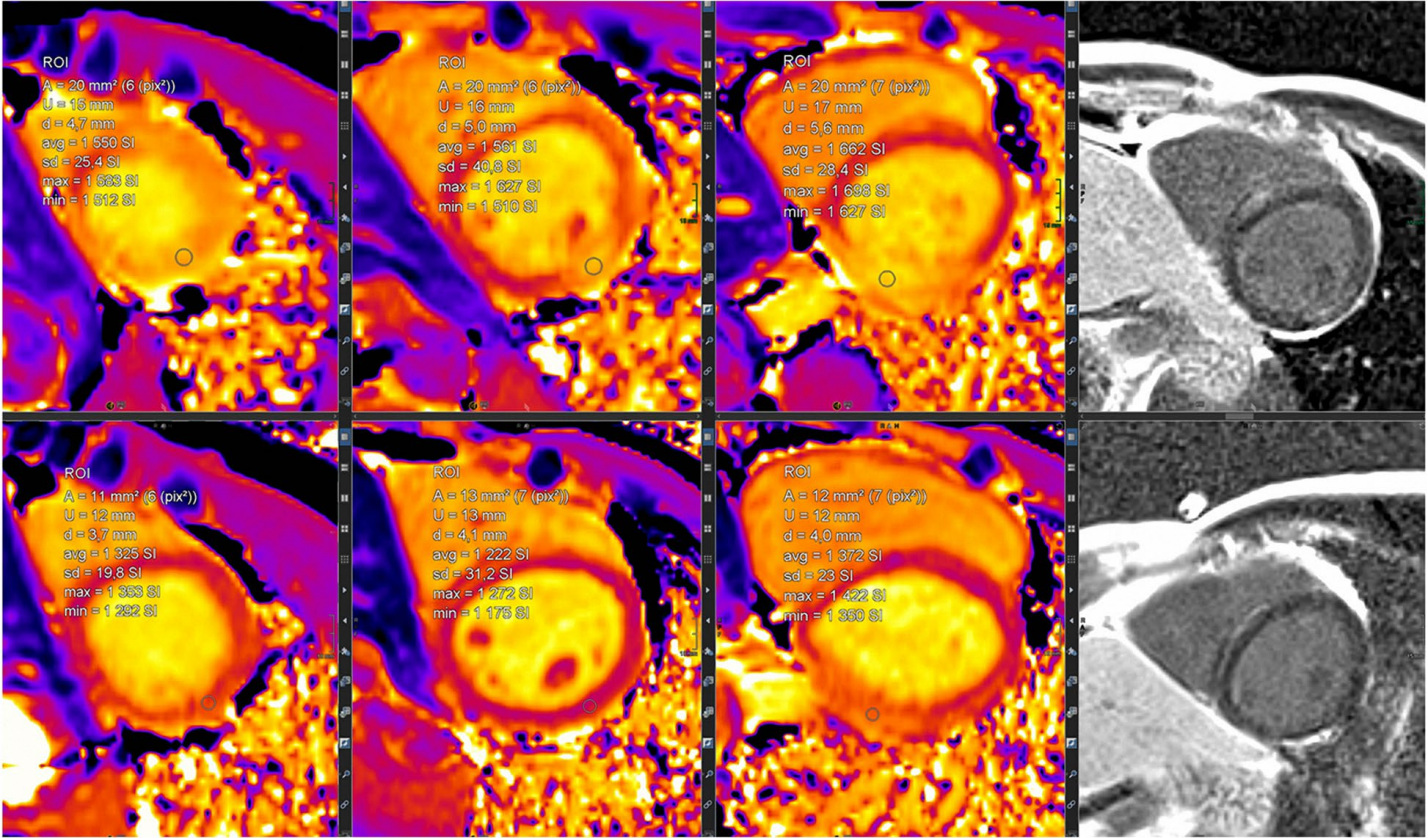
(T1 mapping during follow-up): normalization of initially increased signal intensities in anteroseptal LV myocardium (upper row—baseline with 1550/1561/1662 ms; lower row—follow-up with 1325/1222/1372 ms). The likewise changes of T2 and LGE signal plus T2 as well as T1 maps strongly support the outlined clinical perception

The value of the 2018 Lake Louise criteria for diagnosis and follow-up of adolescent patients has been shown by a study, demonstrating the enhancement of the diagnostic performance adding mapping techniques [27]. Mapping techniques have the potential to predict the length of stay at hospital in acute myocarditis. Moreover, a decrease was noted during follow-up. The decrease of increased T2 signal plus normalization of T2 values is presented in a second case vignette of a 16-year-old adolescent (Figs. 16, 17, and 18). Changes in T2 signal intensity (SI), in mapping, and in LGE were predominantly classically transmurally and subepicardially distinct to the subendocardial pattern in ischemic heart disease. Large multicenter studies like the one from Martins et al. [28] assessing 125 pediatric patients with a clinical diagnosis of acute myocarditis and clinical follow-up in a period of a little bit more than 1 year demonstrated that complete recovery occurs only in 2/3 of patients and that functional recovery was inversely correlated with the presence of certain structural changes according to cardiac MRI (mid-wall or mixed LGE).

#### Pediatric cardio-oncology

Compared to the workup of myocardial inflammation and CHD, the non-invasive assessment of pediatric cardio-oncology is a new emerging field. Toro-Salazar et al. [29] demonstrated that even asymptomatic pediatric cancer patients exposed to anthracycline therapy developed abnormal strain parameters. This was more sensitive compared to the left ventricular (LV) function. A correlation of imaging parameters to inflammatory markers such as matrix metalloproteinase 7 (MMP7) was observed. More sensitive—at least for early stages of anthracycline-induced cardiotoxicity—might be structural parameters as T2 and T1 mapping. Galán-Arriola et al. [30] found that the earliest doxorubicin cardiomyopathy MRI parameter was T2 relaxation time prolongation at week 6, in the absence of T1 mapping, extracellular volume (ECV), or LV motion alterations. The T2 prolongation correlated with intracellular edema. Subsequently, T1 mapping and ECV abnormalities developed and coincided with LV motion defects. This was true from week 10 on. Stop of doxorubicin treatment stopped T2 prolongation and LV motion deterioration, which makes it foreseeable that T2 propagation occurs at a reversible disease stage. Chow and coworkers [31] showed that echocardiography as well as CT and MRI imaging plus blood-based biomarkers are very helpful for primary and secondary prevention strategies in relation to anthracycline-related cardiomyopathy.

#### Emerging techniques—fetal cardiac MRI: gating, resolution, motion compensation

Non-invasive fetal cardiac assessment by MRI is an exciting field that has a new impetus by the advent of different fetal cardiac gating strategies [32]. As ECG as the commonly used gating method in the postnatal period is not applicable in the fetus, alternative techniques have been developed which are presently evaluated for clinical use applying standard sequences. Metric optimized gating (MOG) realizes indirect fetal cardiac gating by iterative adjustment of hypothetical trigger timings [33]. The self-gating method derives the cardiac gating signal from periodic signal variations over the cardiac cycle that are due to altered transverse magnetization in the center of the k-space [34]. Both indirect gating methods depend on specialized software and postprocessing. In contrast, Doppler ultrasound (DUS) gating has the advantages of a direct gating method and is sensitive to heart rate variations similar to ECG. With help of an external DUS sensor placed on the maternal abdomen, the fetal heart action can be captured to generate a gating signal [35].

**Fig. 19:**
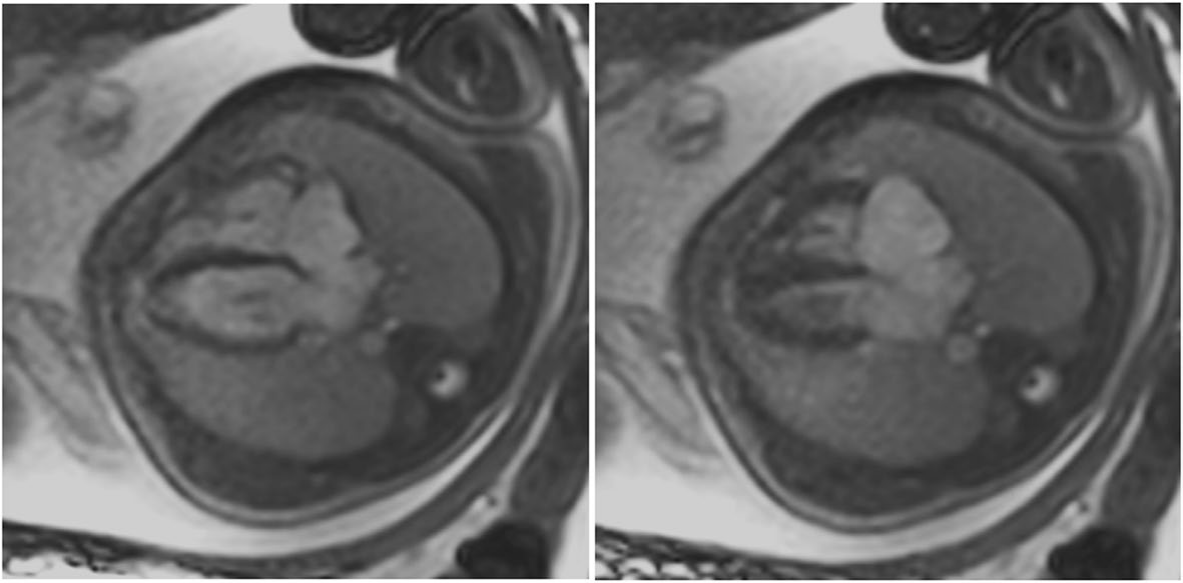
(Four-chamber view in a late gestational healthy fetus): illustrating diastolic (left) and systolic (right) phases using Doppler ultrasound gated cardiac MRI. Ovale foramen as the physiological atrial shunt in the prenatal period is visualized in both diastolic and systolic phases. Motion artifacts (due to missing ECG triggering) normally blur cardiac contours in standard fetal MRI images

**Fig. 20:**
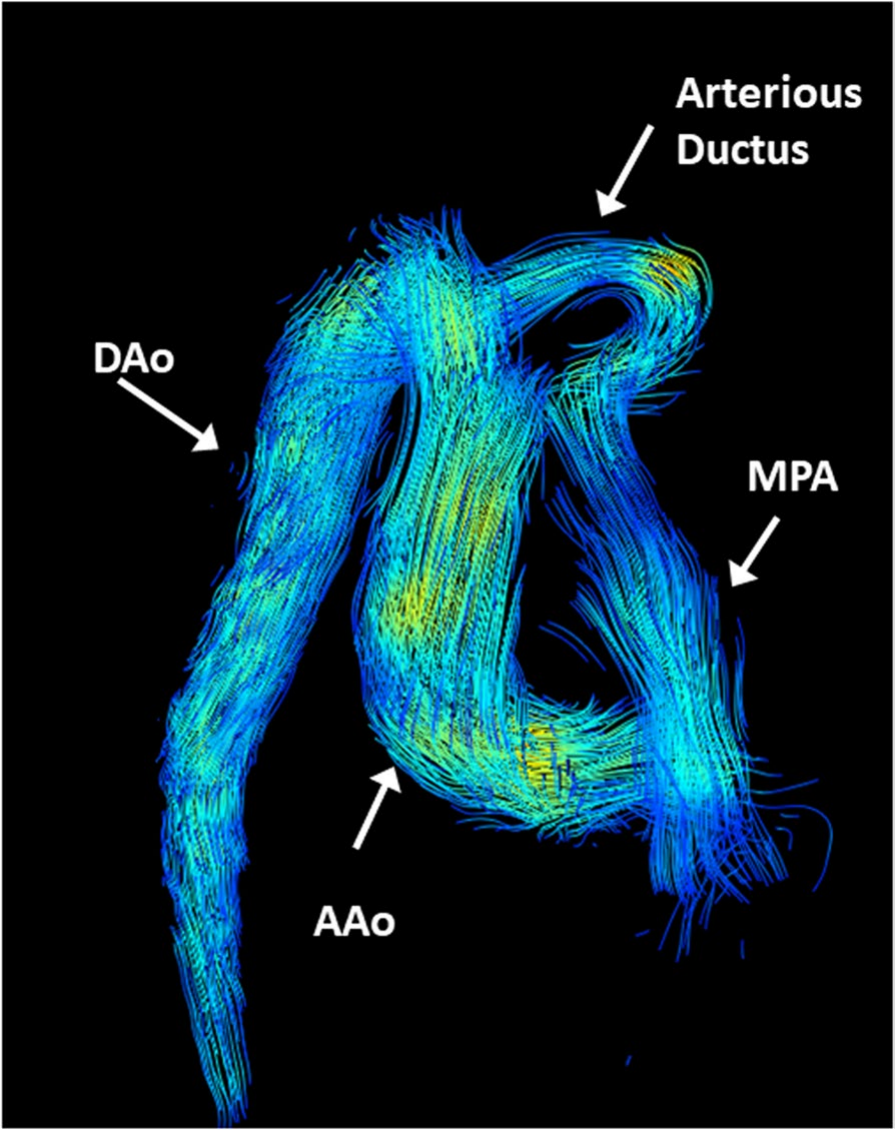
(4D flow MRI): characterization of flow dynamics of the great thoracic vessels (oblique antero-lateral view) in a healthy preterm fetus. Blood flow direction and velocity are indicated by velocity-coded streamlines (DAo descending aorta, AAo ascending aorta, MPA main pulmonary artery, ductus arteriosus). The advent of 4D techniques simplified the demonstration and understanding of even complex vascular anomalies

These gating approaches allow state-of-the-art cardiac imaging with the assessment of cardiac morphology and function, i.e., applying steady-state free precession (SSFP) cine imaging (Fig. 19) or 2D up to 4D flow phase-contrast MR angiography (Fig. 20) for flow assessment [36, 37]. The scan duration of these standard sequences needs to be adapted to allow sufficient maternal breathhold in order to prevent motion artifacts. Considering the small fetal cardiac dimensions and fetal heart rates between 110 and 180 bpm, a high spatio-temporal resolution is required. With the introduction of the aforementioned gating techniques, sufficiently high spatial (i.e., 1 × 1 × 2–5 mm) and temporal (i.e., 12–80 ms) resolutions can be achieved [38]. However, unpredicted fetal motion remains a main challenge for fetal cardiac MRI. Motion compensation techniques are a current field of research to increase the image quality of fetal cardiac MRI [38, 39]. These technical developments may further advance the diagnostic utility of fetal cardiac MRI in the future.

### Artificial intelligence

Data acquisition is one major application for AI-based algorithms [40]. A significant decrease in examination time seems to be possible, at least for MRI. Here, the first commercially available sequences are at hand for certain anatomic areas such as brain imaging and musculoskeletal imaging. It is foreseeable that these imaging techniques will be transferred to cardiac imaging. This will make a major step toward a broad applicability of cardiac MRI for pediatric diseases. The missing link is still the assessment of coronaries by cardiac MRI, where cardiac CT is still the preferred imaging technique. Recent technical improvements like iterative reconstruction were a first major step to reduce dose; however, the use of artificial intelligence will significantly reduce dosages to a higher extent. Recent publications concerning CHD have shown that integrating an AI-based image interpretation might pave the way to new prognostic tools [41].

## Conclusion

Non-invasive cardiac imaging in children and adolescents is one of the pillars of modern diagnostics and therapy planning in inflammatory and structural heart disease and also congenital heart disease. New areas of clinical application are cardio-oncology and fetal cardiac MRI. Available state-of-the-art techniques have decreased the amount of contrast agents and irradiation. Additionally, examination time and also data interpretation time have improved.

Technical developments may help to extend applications of these non-invasive imaging technologies from specialized centers to the broad area. This will be necessary concerning the increasing number of adults with congenital heart disease with normal lifespans. AI-driven technologies will support herein.

## Data Availability

Corresponding author.

## Abbreviations

ASD: Atrial defect
CHD: Congenital heart disease
CINE: Cinematographic imaging
CT: Computed tomography
DGPK: Society for Paediatric Cardiology
DGK: Society of Cardiology
DLP: Dose length product
DRG: Society of Radiology
DSCT: Dual-source CT scanner
ECG: Electrocardiogram
Gd: Gadolinium
IR: Inversion recovery
LGE: Late gadolinium enhancement
LV: Left ventricular
MRA: MR angiography
MRI: Magnetic resonance imaging
PS: Phase-sensitive reconstruction
SAX: Short-axis view
SCMR: Society for Cardiovascular Magnetic Resonance
TCPC: Total cavopulmonary connection
